# An extracellular lipase from *Amycolatopsis mediterannei* is a cutinase with plastic degrading activity

**DOI:** 10.1016/j.csbj.2021.01.019

**Published:** 2021-01-20

**Authors:** Yeqi Tan, Gary T. Henehan, Gemma K. Kinsella, Barry J. Ryan

**Affiliations:** School of Food Sciences and Environmental Health, Technological University Dublin, Grangegorman, Dublin 7 D07 H6K8, Ireland

**Keywords:** Cutinase, Plastic degradation, Comparative modelling, *Amycolatopsis*, Polycaprolactone, Polybutylenesuccinate, Polylactic acid

## Abstract

An extracellular lipase from *Amycolatopsis mediteranei* (AML) with potential applications in process biotechnology was recently cloned and examined in this laboratory. In the present study, the 3D structure of AML was elucidated by comparative modelling. AML lacked the ‘lid’ structure observed in most true lipases and shared similarities with plastic degrading enzymes. Modelling and substrate specificity studies showed that AML was a cutinase with a relatively exposed active site and specificity for medium chain fatty acyl moieties.

AML rapidly hydrolysed the aliphatic plastics poly(ε-caprolactone) and poly(1,4-butylene succinate) extended with 1,6-diisocyanatohexane under mild conditions. These plastics are known to be slow to degrade in landfill. Poly(L-lactic acid) was not hydrolysed by AML, nor was the aromatic plastic Polyethylene Terephthalate (PET). The specificity of AML is partly explained by active site topology and analysis reveals that minor changes in the active site region can have large effects on substrate preference. These findings show that extracellular *Amycolatopsis* enzymes are capable of degrading a wider range of plastics than is generally recognised. The potential for application of AML in the bioremediation of plastics is discussed.

## Highlights

• *Amycolatopsis mediterranei* lipase (AML) exhibits cutinase-like structural features.
• AML shows 60–70% sequence similarity to a few plastic degrading cutinases.
• AML has the ability to degrade poly(caprolactone) and poly(butylene succinate).

## Graphical abstract

**Figure:**
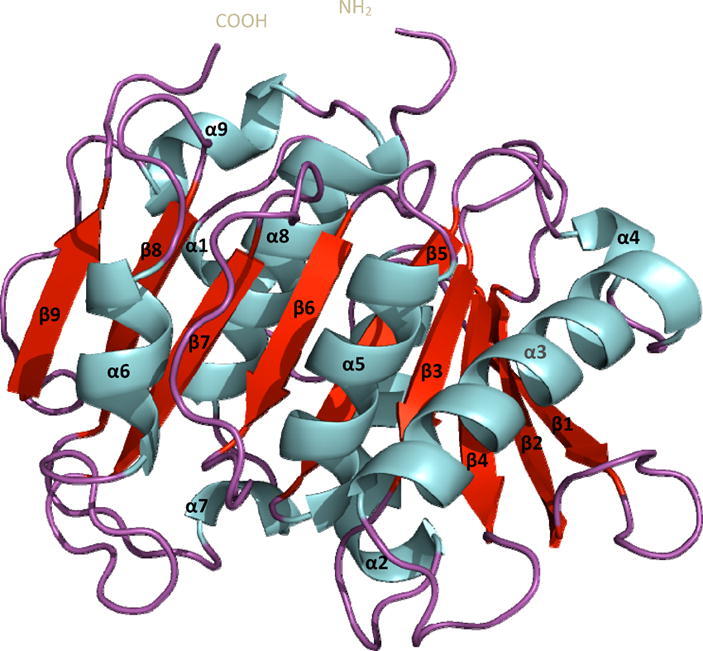

## 1 Introduction

Cutinases are similar to lipases and esterases in their ability to hydrolyse carboxylate esters. They belong to the α/β hydrolase superfamily with the signature α/β fold and a catalytic triad formed by three residues (a nucleophile, histidine, and a catalytic acid) at the active site [1]. Some α/β hydrolases, including lipases, have a ‘lid’ domain that covers the substrate-binding site which ‘opens’ at a lipid-water interface [2].

In previous work, we identified an interesting extracellular lipase from *Amycolatopsis mediterannei* (AML) with biotechnologically useful properties [3]. In the present study, we examine the three-dimensional structure of AML by comparative modelling. This modelling approach uses experimentally determined structures of homologous proteins as templates [4]. A template with > 50% similarity to the target can provide good quality predictions, often as good as X-ray elucidated low resolution structures [5]. Lipases have previously been structurally characterised using comparative modelling techniques [6].

The structural characterisation of AML along with substrate specificity studies show that this enzyme is more appropriately described as a cutinase. A BLAST search of the α/β hydrolase database found that AML was homologous to several known plastic degrading enzymes. When tested for this activity, AML was found to be capable of the degradation of certain aliphatic, but not aromatic, plastics. This interesting specificity is discussed in terms of the active site structure and homology with other plastic degrading enzymes. The ability of AML to degrade aliphatic plastics underlines the fact that *Amycolatopsis* is capable of digesting a wider range of polyesters than is generally recognised. The significance of an extracellular plastic degrading cutinase in *Amycolatopsis* species is discussed.

## 2 Materials and methods

### 2.1 Materials

pET-49b (+) plasmid was from Merck Novagen®; Macherey-Nagel™; Protino® Glutathione Agarose was obtained from Fisher Scientific and all other chemicals were from Sigma Aldrich.

### 2.2 Production of AML

AML was produced using the recombinant system pET-49b (+) in *Escherichia coli* BL21(DE3) cytosol as described by Tan et al. (2021; in press). A starter culture (MDG media; [7]) was inoculated and incubated overnight at 37 °C and 300 rpm. The starter culture was added at a 1:1000 ratio to the culture media and incubated at 37 °C and 300 rpm until the OD_600_ reached 0.5–0.7. At this point, IPTG was added to a final concentration of 1 mM to induce expression. The induced culture was incubated at 25 °C overnight and centrifuged at 13,000 × *g* for 2mins at 4 °C. The pellet was resuspended in 3 ml per 20 ml culture of lysis buffer and was sonicated (Q55 Sonicator with a standard 1/8″ diameter probe from QSonica, LLC) on ice using 30–60 s bursts at a setting of 30%-40% amplitude. The sonicated mix was pelleted at 12,000 × *g* for 15mins. The supernatant was loaded on a Macherey-Nagel™ Protino® Glutathione Agarose column and AML was cleaved off from the tag using GE Healthcare PreScission® Protease.

### 2.3 Comparative modelling of AML

The amino acid sequence of the AML was obtained from the National Centre for Biotechnology Information (NCBI) database (*Amycolatopsis mediterranei* U32 lipase GenBank accession no. ADJ49206). The sequence was inputted into the interactive web interface of SWISS-MODEL (https://swissmodel.expasy.org/interactive). The 3-D template coordinates of *Streptomyces exfoliatus* lipase (PDB code: 1jfr; [8]) were chosen and using a target-template sequence alignment an all-atom model of the AML target sequence was generated using ProMod-II [9]. The initial quality assessment of the generated model used the information provided by SWISS-MODEL: target-template alignment, step-by-step modelling log, oligomeric state, ligands, and cofactors in the model.

### 2.4 Model quality validation

The quality of the model coordinates was evaluated using the QMEAN score of the SWISS-MODEL server and the tools of the Structure Analysis and Verification Server (SAVES) (http://servicesn.mbi.ucla.edu/SAVES/), including VERIFY3D [10] and ERRAT, [11] Protein Structure Analysis (ProSA) server (https://prosa.services.came.sbg.ac.at/prosa.php; [12]), and RAMPAGE (http://mordred.bioc.cam.ac.uk/≃rapper/rampage.php; [13]). The secondary structure of AML was predicted using JPred4 (http://www.compbio.dundee.ac.uk/jpred/; [14]). The visualisation and figure generation were performed using PyMOL v2.3.3 [15].

### 2.5 Classification of AML and identification of its functional residues

The amino acid sequence of AML in FASTA format (GenBank ID: ADJ49206) was searched through the BLAST tool [16] available on the LED database (http://www.led.uni-stuttgart.de/) and ESTHER database (http://bioweb.supagro.inra.fr/ESTHER/general?what = blast) to classify AML according to the family system in each database respectively.

### 2.6 Flexible docking of substrate onto AML model

All the protein and ligand structures were prepared for docking using Biovia Discovery Studio 4.0 [17]. The binding site was defined by a sphere encompassing corresponding residues in Table 1. Flexible docking was performed with Biovia Discovery Studio 4.0, using default parameters and setting the specific residues as flexible. The visualisation and figure generation were performed using PyMOL Molecular Visualisation System v2.3.3 [15].

**Table 1:** List of rigid and flexible binding site residues defined for ligand docking.

Rigid residues	Flexible residues
110–112,142,177,179,203,257 and 260.	178, 224 and 256

### 2.7 Turbidity assay for aliphatic plastic degradation

The turbidity assay for polyester degradation was carried out as outlined in Masaki and colleagues, [18] with slight modification. The enzyme degradation of plastics was carried out at 30 °C with continuous shaking at 50 rpm for up to 2 days. One gram of polyester (Merck Sigma-Aldrich Catalogue#: 448028; poly(1,4-butylene succinate), extended with 1,6-diisocyanatohexane, 440,752 (polycaprolactone), or GF45989881 (polylactic acid)) was dissolved in 6 ml of chloroform with the aid of a water bath sonicator, along with 100 mg of Plysurf A209G surfactant (DKS Co. Ltd.). The solution was added to 25 ml of water and shaken vigorously to create an emulsion. The medium was autoclaved at 121 °C at 15psi for 20mins to evaporate the chloroform. The final reaction mixture contained 0.04% w/v emulsified plastic, 0.0016% (w/v) Plysurf A209G, 50 mM Tris HCl (pH8.0) and AML (2.5µg/ml). The degradation ratio was calculated by measuring the decrease in turbidity at 660 nm of solutions before and after the addition of the enzyme, with a reading being taken at one-hour intervals for 6hrs.

### 2.8 PET degradation assay

The ability of AML to degrade PET was tested using the procedure adapted from Austin and co-workers [19]. The PET film of 2.5 μm thickness (Goodfellow Cambridge Ltd. Catalogue#: ES301025) was placed in a 1.5 ml Eppendorf tube with 300 µL of 50 mM phosphate buffer (pH8.0) and with 2 µM AML. The digestion was carried out at 30 °C with agitation at 150 rpm. After 72hrs of digestion, the reaction was terminated by enzyme inactivation by heating at 85 °C for 15 min. The AML treated films were rinsed with 1% (v/v) SDS, followed by dH_2_O, and then ethanol and left in a 37 °C incubator to dry overnight (18hrs). Subsequently, the sample was centrifuged at 17,000 × *g* for 10 min. The films were coated with a 6 nm layer of gold/palladium (Au/Pd) and analysed using a Hitachi SU6600 Field Electron Microscopy (FESEM) instrument employed at a 5.0 kV voltage acceleration, with a 7.1 mm working distance and beam current of 17µA.

The supernatant of AML-treated PET film was removed for HPLC and fluorescence spectroscopy analysis. Terephthalic acid (TPA) and bis(2-Hydroxyethyl) terephthalate (BHET) released from PET degradation were quantified using HPLC. The HPLC analysis was performed using a Waters Alliance e2695 Separations Module equipped with ZORBAX Eclipse XDB C-18 (80 Å, 5 µm, 4.6 mm × 150 mm) HPLC column from Agilent Technologies. The mobile phase was neat methanol/50 mM phosphate-citric acid buffer (60:40% (v/v), pH3.8). Standard solutions of TPA and BHET over a concentration range of 0.05 – 1.0 mM were prepared as the reference for sample detection. The elution was operated at the flow rate of 1.0 ml/min in isocratic mode. The eluent peaks were detected at 241 nm using a Waters Alliance 2998 Photodiode Array Detector.

The analysis was performed as per Silva and Cavaco-Paulo [20] and Nimchua and colleagues, [21] with minor adaptations. The terephthalic acid released from PET degradation was quantified by fluorometric determination at 425 nm from hydroxy-terephthalic acid (λ_eλ_ = 315 nm, λ_em_ = 425 nm) formed by the reaction of TPA and hydrogen peroxide. The reaction was performed by adding 2 ml of hydrogen peroxide into 1 ml of the reaction aliquot. The mixture was heated at 90 °C for 30mins and the reading was taken after the samples had cooled to room temperature. A calibration curve was plotted using the reading from standard solutions of TPA (0.006, 0.03, 0.06, 0.12 mM) dissolved in 0.05 M NaOH solution.

## 3 Results and discussion

### 3.1 Modelling studies

**Fig. 1:**
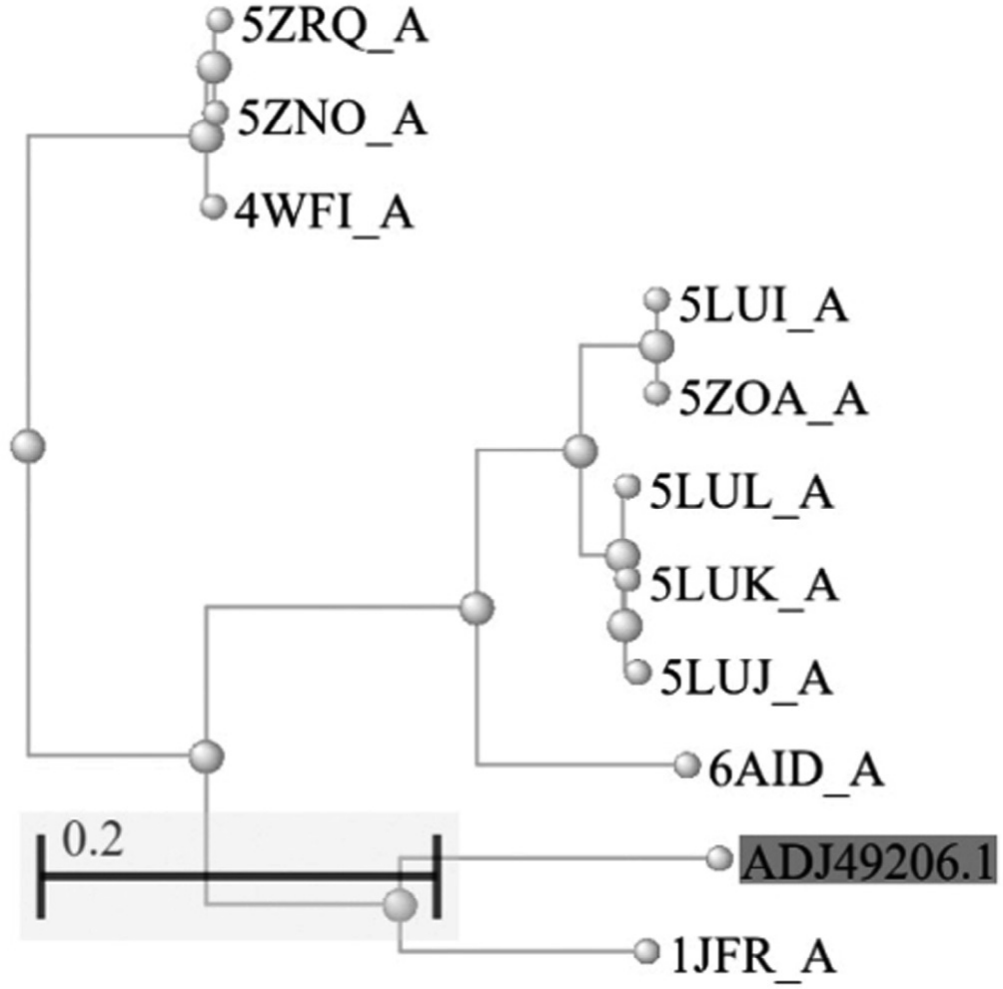
Phylogenetic tree of AML (highlighted in grey; GenBank ID: ADJ49206.1), *Saccharomonospora viridis* cutinase S176A/S226P/R228S mutant (PDB ID: 5ZRQ_A), *Saccharomonospora viridis* cutinase S176A/S226P/R228S mutant (PDB ID: 5ZNO_A), with *Saccharomonospora viridis* cutinase (PDB ID: 4WFI_A), *Thermobifida cellulosilytica* cutinase 1 (PDB ID: 5LUI_A), BTA_hydrolase 1 from *Thermobifida fusca* (PDB ID: 5ZOA_A), *Thermobifida cellulosilytica* cutinase, triple variant (PDB ID: 5LUL_A), *Thermobifida cellulosilytica* cutinase 2, double variant (PDB ID: 5LUK_A), *Thermobifida cellulosilytica* cutinase 2 (PDB ID: 5LUJ_A), *Thermobifida alba* cutinase Est119 (PDB ID: 6AID_A), and *Streptomyces exfoliatus* lipase (PDB ID: 1JFR_A). The tree of the first 10 BLASTp hits was constructed using pairwise alignment in BLASTp [16] with the setting of “Fast Minimum Evolution” tree method, “Grishin (protein)” distance model, and a maximum sequence distance of 0.4.

The template for comparative modelling of AML was selected through a BlastP search against the Protein Data Bank (PDB) database. A phylogenetic tree was constructed to find the available templates with the closest evolutionary relationship to AML (Fig. 1). AML was most closely related to a lipase from *Streptomyces exfoliatus* sharing 75% sequence identity, which is, therefore, suitable as a template for modelling studies [22].

A comparative model of AML (262 residues, sequence 47–309) was constructed using the selected *Streptomyces exfoliatus* (PDB: 1JFR) template with the SWISS MODEL server. The developed AML model was evaluated through a variety of validation tools including RAMPAGE, Verify3D, ERRAT and ProSA (see supplementary Figs. 1-4 and Table 1) and findings are summarised in Table 2.

**Table 2:** Summary table of the quantitative analysis for the model quality of AML and the *S.exfoliatus* lipase template (PDB ID: 1JFR).

Qualitative measures	Threshold value	AML	1JFR
Ramachandran plot % residues in favoured region (~98% expected)	~98%	98.1%	98.4%
Verify3D Average 3D-1D score	>80%	97.3%	98.5%
ERRAT % protein below 95% limit	95% (high resolution)91% (low resolution)	86.7%	94.8%
ProSA Z-score	n/a	−6.9	−7.3

Ramachandran plots [13] assess the stereochemical quality of the model, which compared favourably (~98.0% of residues in the favoured region and 2% in the allowed region) with the template structure. The structural integrity of the model was first evaluated using Verify3D, [23] which determines the compatibility of an atomic model (3D) with its own amino acid sequence (1D). A Verify3D result of 97.3% indicates that there is a good folding quality to the AML model. ERRAT [11] analyses the statistics of non-bonded interactions between different atom types and plots the value of the error function versus position. According to the ERRAT plot of AML, 87.7% of the residues fall below the 95% rejection limit of incorrect non-bonded interaction. Finally, ProSA [12] identifies errors in the 3D structure of proteins. The resultant Z-score of AML and of the 1JFR template both fall within the range for similarly sized native proteins and both energy profile plots show a good overall local model quality. In summary, the overall model quality of AML is very close to the 1JFR template and has a similar quality profile to recent homologous models of lipases reported in the literature [24], [25].

Through sequence alignment with the template, the catalytic triad and the AML oxyanion forming residues were identified as Ser178, Asp224, His256 and Phe110, Met179 respectively. Ser178, Asp224, and His256 are the triad responsible for the hydrolysis of the acyl group while the amide backbones of Phe110 and Met179 help to stabilise the enzyme-substrate intermediate via H-bonding with the carbonyl oxygen of the substrate [26]. The catalytic triad and the oxyanion residues form part of the substrate binding pocket (see supplementary Fig. 5). The alignment also showed that the enzyme lacks a lid domain, similar to the template structure used [8]. The absence of a lid domain has been reported in several plastic degrading cutinases [27].

**Fig. 2:**
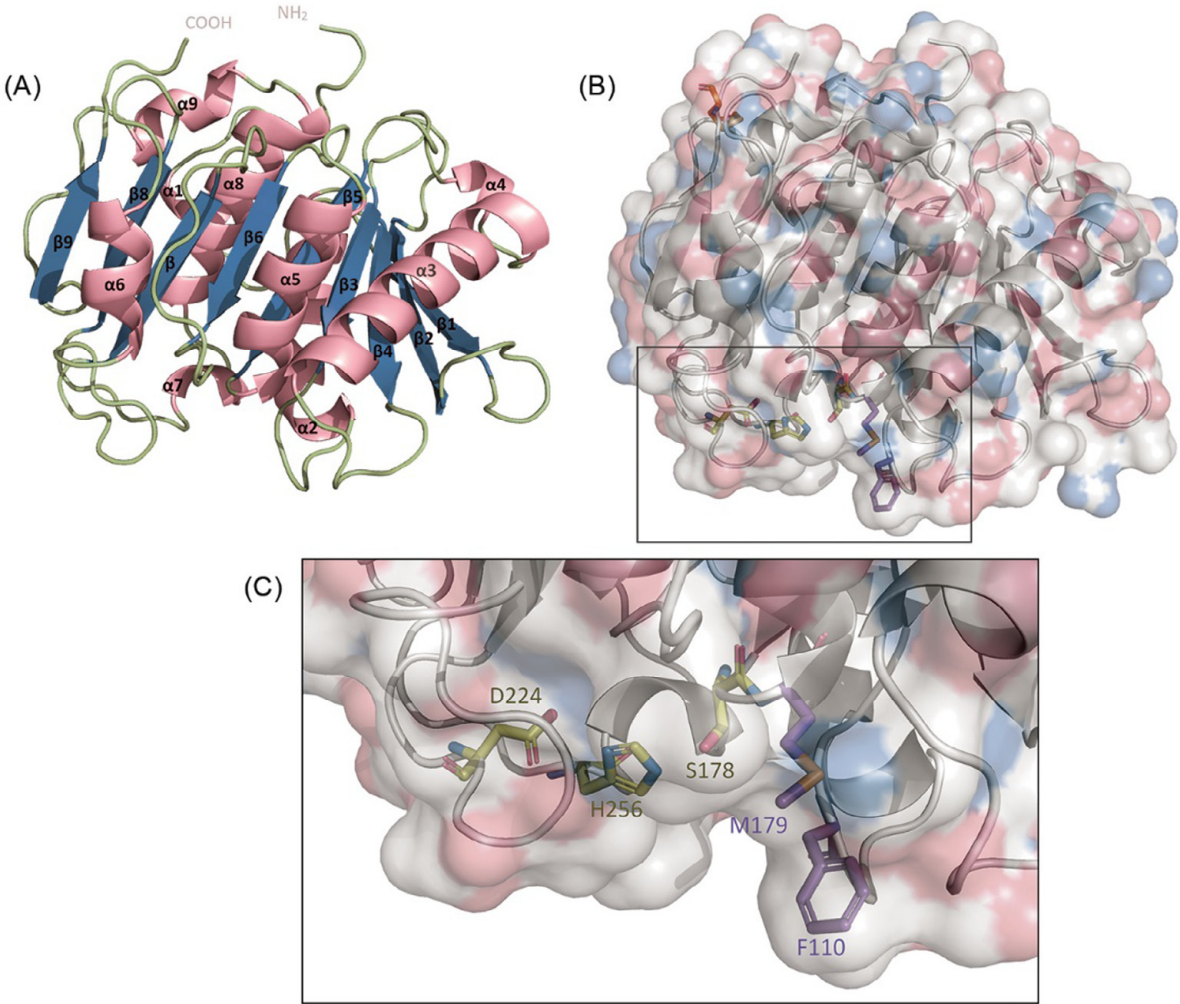
(A) The 3D homology model of AML generated using the crystal structure of lipase from *Streptomyces exfoliates* (PDB ID: 1JFR) as a template through the SWISS-MODEL server. The *β*-sheet is shown in blue and α-helices are shown in pink while loops are shown in a pale green colour. (B) Surface model of AML displaying the essential active site residues. (C) The catalytic triad (yellow) and oxyanion hole residues (purple) are shown as stick models. The white, blue and red colours on the surface model represent carbon (C) atoms, oxygen (O) atoms, and nitrogen (N) atoms respectively. The figure was generated using PyMOL Molecular Visualisation System v2.3.3 [15]. (For interpretation of the references to colour in this figure legend, the reader is referred to the web version of this article.)

The AML model has the typical α/β hydrolase structure – with a central β sheet surrounded by α-helices (see Fig. 2A). Furthermore, a disulphide bond has been modelled between Cys289 and Cys305. The location and the conformation of the functional residues were investigated using the 3D model of AML. It can be seen that the functional residues formed part of a potential substrate binding pocket below the β-sheet (see Fig. 2B & C).

**Fig. 3:**
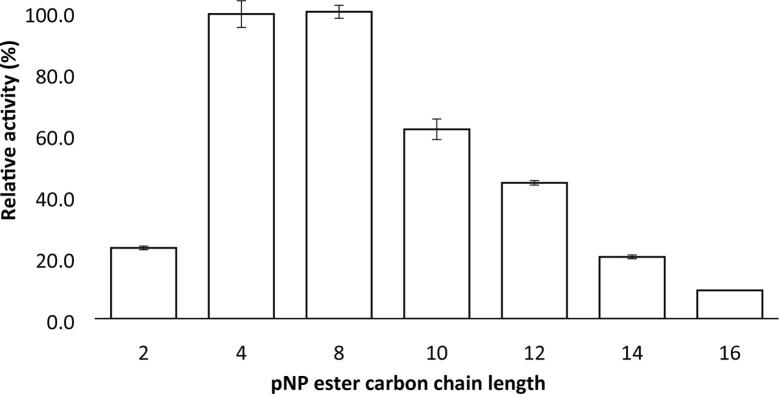
Experimentally determined substrate specificity of AML for *p*-nitrophenyl (*p-*NP) esters of various carbon chain lengths (C-2 to C-16). All substrates were prepared at 20 mM in 50 mM Tris buffer (pH7.5) and assayed at a temperature of 37 °C for 10 min in triplicate as described previously [3]. The relative activity (%) was calculated using *p*-nitrophenyl octanoate (C-8) as the reference, highest activity, substrate.

**Fig. 4:**
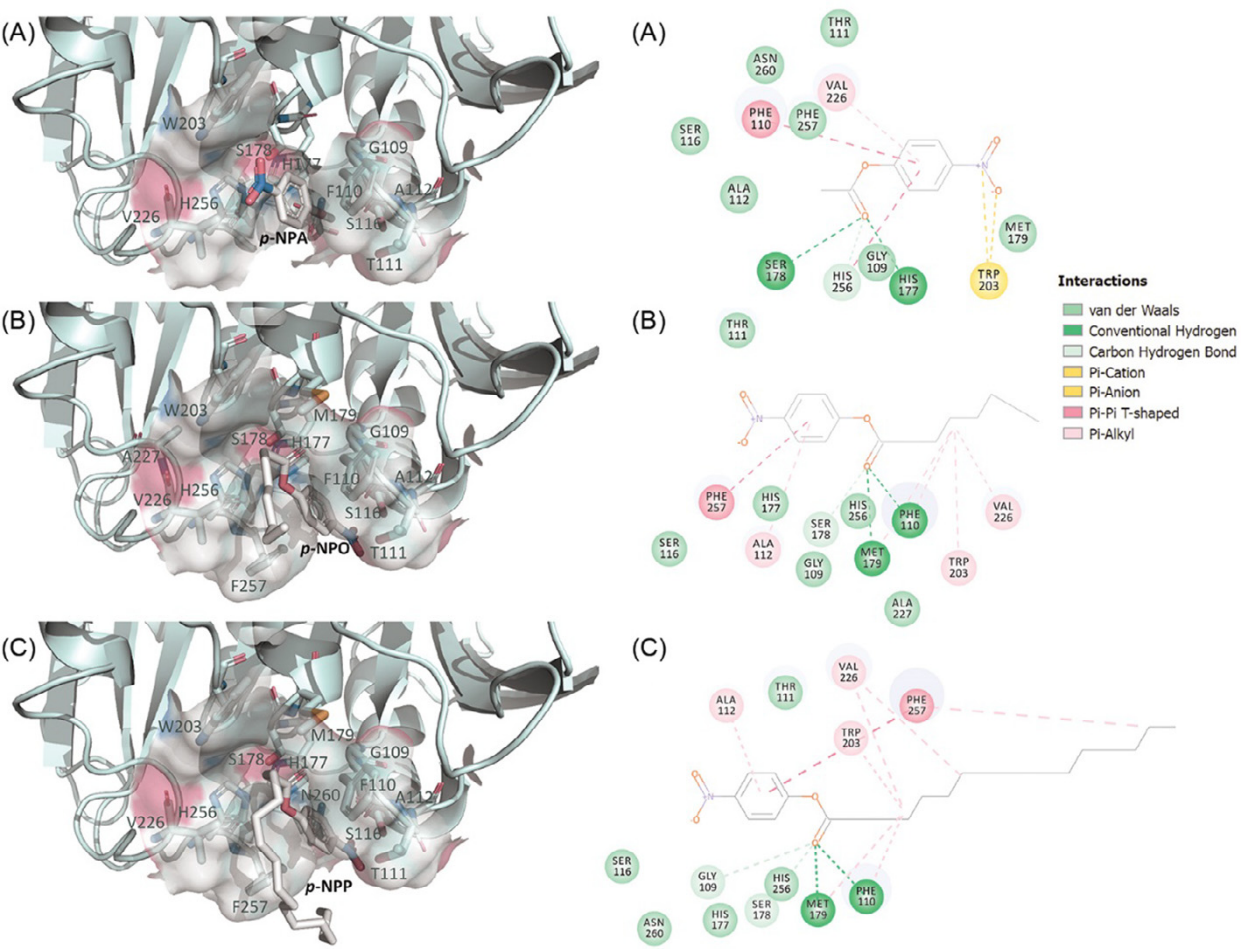
3D-diagram demonstrating the docking of (A) *p*-nitrophenyl acetate (*p*-NPA), (B) *p*-nitrophenyl ocatanoate (*p*-NPO), and (C) *p*-nitrophenyl palmitate (*p*-NPP) in the developed AML model with their corresponding 2D interaction maps. The residues with predicted non-covalent interactions are shown as stick and surface models. The ligands were displayed as white stick model while AML was shown in cartoon format . The 3D diagrams were generated using PyMOL v2.3.3 (DeLano Scientific, 2002) and the 2D interaction maps were generated using Discovery Studio Visualiser [17].

The experimentally determined preference of AML for medium chain length fatty acids (Fig. 3) matches the known preference of cutinases which tend to favour substrates with acyl chain lengths of 4–8 carbons. This indicates that AML is closer to a cutinase type lipase rather than an esterase or true lipase [28], [29]. AML has a binding site with a similar shape to a typical lipase binding site, but with a smaller hydrophobic region for fatty acid chain binding.

To inspect how substrates of different length are predicted to bind to AML, the docked poses of AML with *p*-nitrophenyl acetate (C-2), *p*-nitrophenyl octanoate (C-8), and *p*-nitrophenyl palmitate (C-16) were generated using flexible docking in Biovia Discovery Studio 4.0. The binding poses were visualised using PyMOL and the ligand protein interactions are shown in a 2D map generated with Biovia Discovery Studio (Fig. 4).

The docked structures of AML with the C-8 and C-16 substrates have their acyl groups bound to the predicted hydrophobic binding site. The 2D interaction maps also showed that the carbonyl oxygen of C-8 and C-16 form hydrogen bonds with the backbone amide group of the oxyanion residues of AML (Phe110 and Met179) required for tetrahedral intermediate stabilisation during catalysis [30]. The main hydrophobic residues that are predicted to interact with the fatty acid chain of the acyl group are Gly109, Phe110, Met179, Trp203, and Val226. In contrast, for *p*-nitrophenyl acetate the acyl group is pointed towards the less hydrophobic part of the binding pocket and instead has predicted hydrogen bond contacts with His177 and Ser178.

*p*-nitrophenyl acetate has the acyl group pointed towards the less hydrophobic part of the binding pocket. This resulted in a suboptimal interaction of the carbonyl group with the oxyanion residues and the catalytic His225, which is important for the release of an alcohol group during the hydrolysis process [31]. This might explain the experimentally low activity of AML observed towards *p*-nitrophenyl acetate .

The main hydrophobic residues that interacted with the fatty acid chain of the acyl group are Gly109, Phe110, Met179, Trp203, and Val226. While Gly109 and Trp203 interact with C1 of the acyl group, Phe110 and Met179 interact with C2 and C3 of the acyl group and the position of their backbone amide is also important for intermediate stabilisation. Val226 interacts with C4 (C-8 substrate) and C5 (C-16 substrate), indicating that the mutation of Val226, to a bulkier hydrophobic residue, could shift the substrate preference of AML towards shorter acyl chains. Such engineering of a lipase substrate binding site for short acyl length preference has been previously demonstrated for *Candida rugosa* LIP4 [32] and *Candida antartica* lipase A [33].

### 3.2 Plastics degradation

AML was found to be homologous to several plastic degrading cutinases (see Table 3). The ability of AML to degrade polyesters was tested on common, commercially- used, plastics as substrates: aliphatic polyesters poly(butylene succinate), poly(caprolactone), poly(lactic acid) and the aromatic poly(ethylene terephthalate).

**Table 3:** Polyesterase-lipase-cutinase family proteins that are homologous to AML and have reported polyester degrading ability. Y or N denotes if a structure is available in the structural databases or not.

Protein	Polyester(s) substrate	GenBank	Sequence identity/similarity to AML (%)	Structure	Reference(s)
*Streptomyces* sp. SM12 SM14 Polyethylene Terephthalate Hydrolase (PETase)-Like Enzyme	Polycaprolactone	WP_103503499	57/73	N	[49]
*Thermobifida alba* AHK119 Esterase Est119	Apexa® 4026Polylactic acidPoly(ethylene terephthalate)	BAI99230.2	61/73	Y	[50], [51]
*Thermobifida cellulosilytica* Cutinase 2	Polylactic acidPoly(ethylene terephthalate)	ADV92527.1	62/76	Y	[28], [52], [53]
Leaf and branch compost cutinase (LCC)	Poly(ethylene terephthalate)	AEV21261.1	57/74	Y	[29], [54], [55]
*Ideonella sakaiensis* (strain 201-F6) Poly(ethylene terephthalate) hydrolase	Poly(ethylene terephthalate)	GAP38373.1	49/67	Y	[19], [44], [45], [56]
*Saccharomonospora viridis* PET-degrading cutinase Cut190	Poly(ethylene terephthalate)	BAO42836.1	63/78	Y	[57], [58], [59], [60]
*Thermobifida cellulosilytica* Cutinase 1	Poly(ethylene terephthalate)	ADV92526.1	63/78	Y	[28]

### 3.3 Aliphatic polyester plastics

**Fig. 5:**
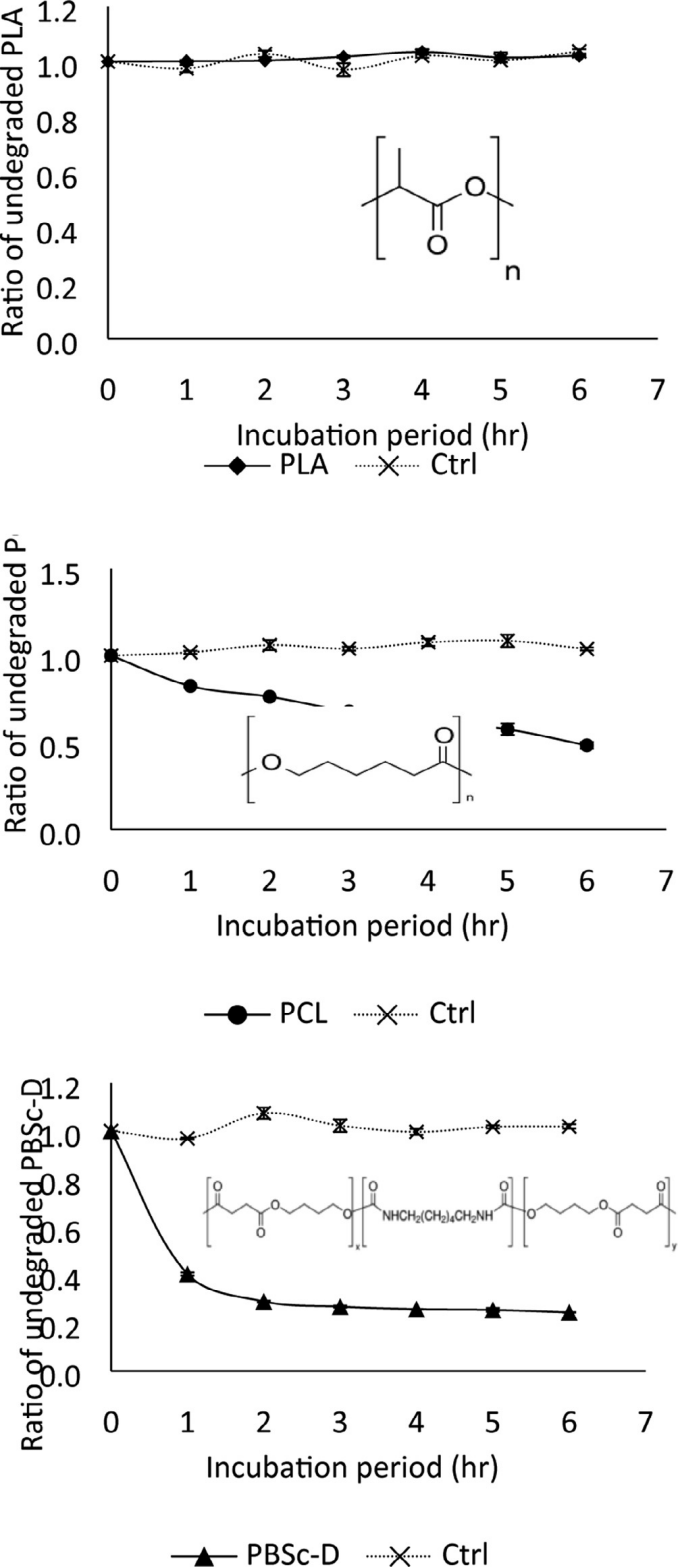
Degradation of PLA (♦), PCL (●), and PBSc-D (▲) by AML. The degradation was carried out with a final enzyme concentration of 2.5 µg/ml AML with incubation at 30 °C in 50 mM phosphate buffer (pH 8.0) in duplicate. A reaction mixture incubated without any enzyme was used as a control (**×**). The ratio of undegraded polyesters was calculated using the ratio of A_660_ after the incubation period with the initial A_660._

Poly(lactide; PLA) is composed of lactic acid monomers. It is a widely used plastic and is the fastest growing market of all bioplastics [34]. It is known to be quite resistant to biodegradation in the environment [35]. Enzymatic breakdown of PLA by AML over a short time period (6hrs) at moderate temperature (30 °C) was not successful (Fig. 5). This was somewhat surprising since the extracellular enzymes of *Amycolatopsis* species have previously been described as having PLA degrading activity [36]. Conversely, under the same conditions, AML degradation of poly(caprolactone) was more successful, with almost 60% degradation observed following 6 h of AML exposure (Fig. 5). Poly(caprolactone) is a bioplastic extensively used in controlled release drug formulations and is only degraded by a limited number of enzymes [37]. Of the three aliphatic polyesters examined, AML was most successful in the degradation of poly(1,4-butylene succinate) extended with 1,6-diisocyanatohexane (PBSc-D) under the conditions explored (Fig. 5). poly(butylene succinate; (PBSc-D) is a petroleum derived plastic which is considered among the more persistent plastics in the environment [38], [39]. This enzyme may be a useful tool for bioremediation of this rapidly growing class of environmental plastics.

The effect of degradation over time was also examined. Extensive hydrolysis by AML was observed with PCL and PBSc-D as substrates when the hydrolysis of polyester plastics was carried out for an extended time period (22hrs) at a modest temperature (30 °C): these polymers were degraded by 90 and 80% respectively Table 4).

**Table 4:** Table detailing a comparison of the % of degraded PCL, PLA and PBSc-D by AML at incubation periods of 0, 6, and 22hrs at 30 °C in 50 mM phosphate buffer pH 8.0.

Incubation period (hr)	% of degraded polyester		
	PCL	PLA	PBSc-D
0	0	0	0
6	50	0	80
22	90	0	80

### 3.4 Strucutral and sequence analysis to predict PLA degradation

The mechanism of PLA degradation by a cutinase from *T. alba* has been recently reported [40]. The cutinase (*T.alba* cutinase Est119) has a sequence similar to AML (61% sequence identity, 73% sequence similarity). In view of the observed lack of PLA degradation by AML, its active site topology compared to Est119 was examined. The objective was to try to understand the differences between these related plastic degrading enzymes at a structural level.

**Fig. 6:**
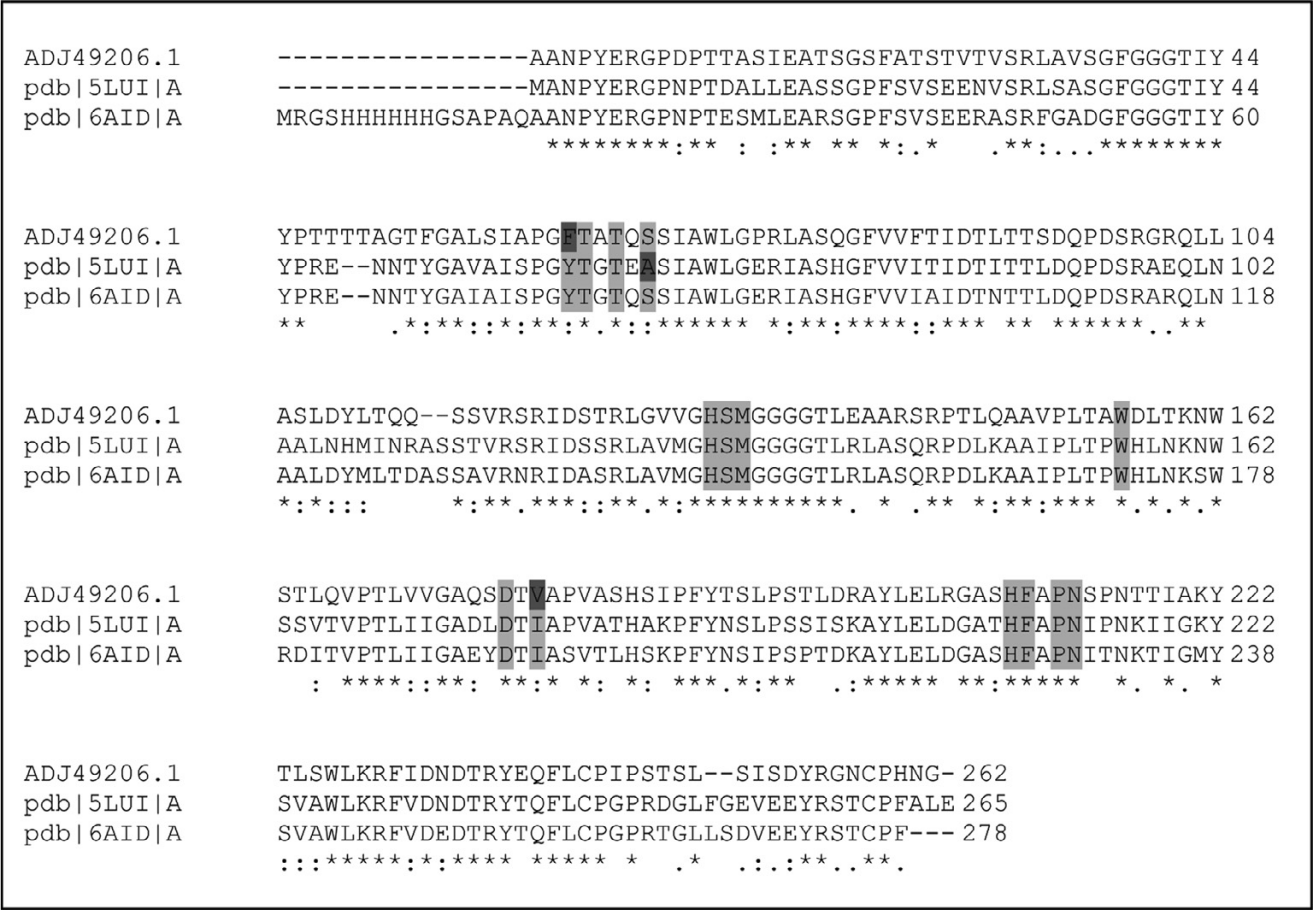
Multiple sequence alignment of AML (Accession code: ADJ49206) with homologous cutinases which were reported to have polylactic acid degrading properties in the literature: cutinase from *Thermobifida cellulosilytica* (PDB ID: 5LUI) and Est119 from *Thermobifida alba* AHK119 (PDB ID: 6AID). The key substrate binding residues were highlighted in light grey with differences between the enzymes residues highlighted in dark yellow. The alignment was performed using the Clustal Omega server (https://www.ebi.ac.uk/Tools/msa/clustalo/). (For interpretation of the references to colour in this figure legend, the reader is referred to the web version of this article.)

**Fig. 7:**
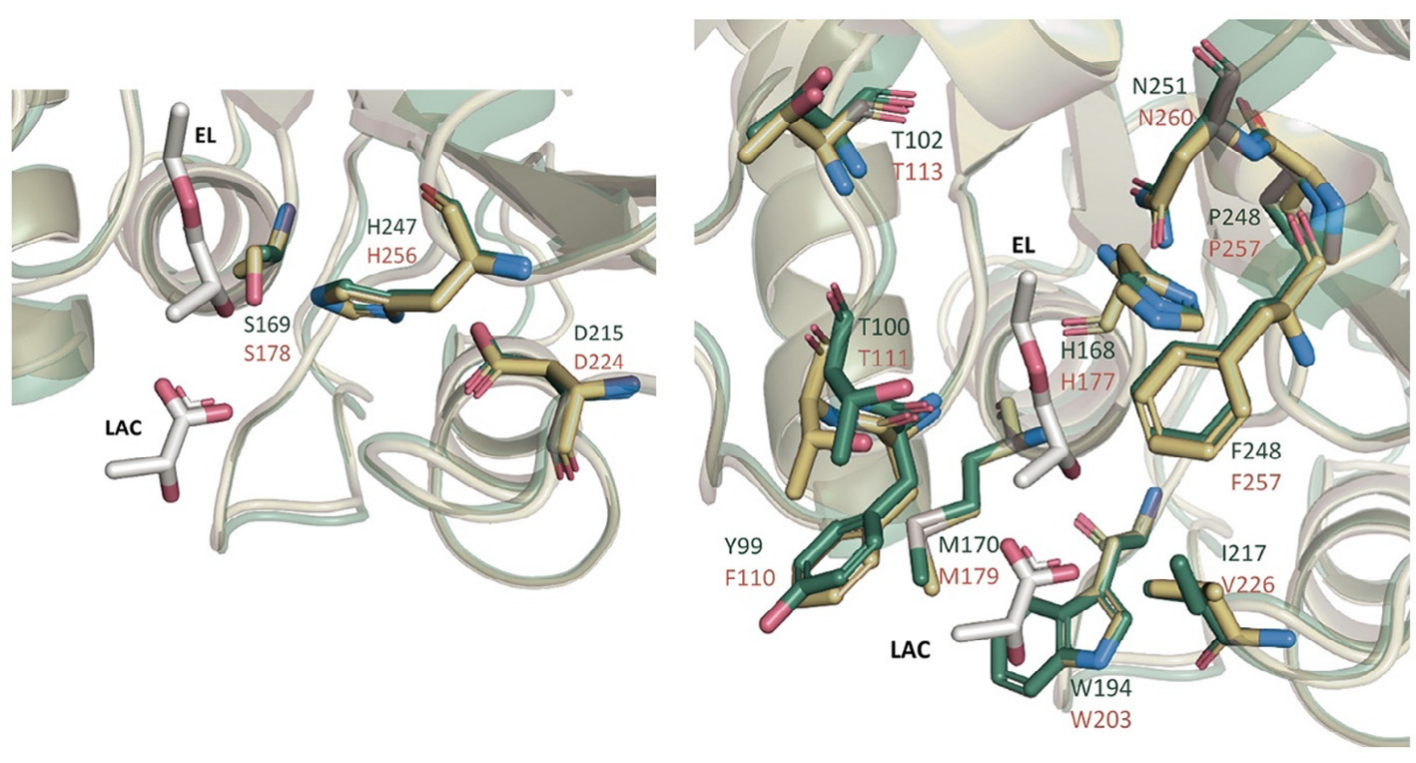
On the left, the structural comparison of AML (mustard yellow) with PLA degrading *T.alba* cutinase Est119 (green; PDB: 6AID) focusing on the catalytic residues. The superposition was performed in PyMol using C_α_ atoms with default parameters. The catalytic triad residues, shown as stick models H 247/256; D 215/224 and S169/178 are seen to overlap in this model. Ethyl lactate (EL) and lactic acid (LAC) were presented as stick models in white. On the right, is the structural comparison of AML (mustard yellow) with PLA degrading *T.alba* cutinase Est119 (green; PDB: 6AID) showing residues of the substrate recognition site, subsites I and II. (For interpretation of the references to colour in this figure legend, the reader is referred to the web version of this article.)

The amino acid sequences of AML, Est119 and another PLA degrading cutinase from *Thermobifida cellulosilytica,* were aligned to examine the difference in key substrate binding residues between them (Fig. 6). It was clear from this alignment that there was significant overlap in the key catalytic residues for these enzymes. The structural differences were further explored by superimposing the 3D model of AML on the crystal structure of Est119 crystallised with bound PLA analogues ethyl acetate (EL) and lactic acid (LAC; PDB: 6AID) [40]. Both structures were aligned using PyMOL to compare the reported catalytic and substrate recognition sites for PLA degradation (see Fig. 7).

**Fig. 8:**
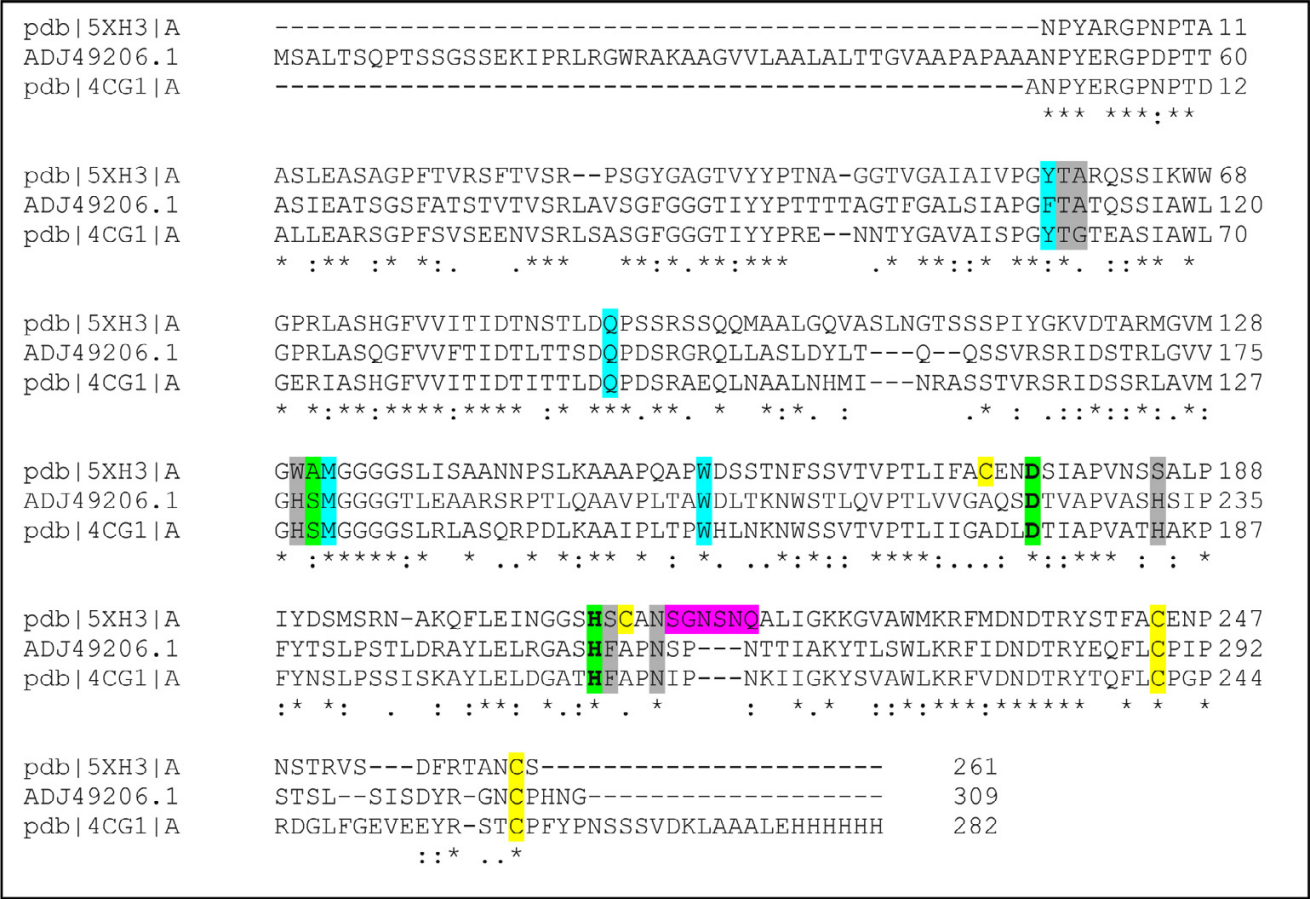
An alignment of the amino acid sequences of AML (GenBank ID: ADJ49206) with PETase from *Ideonella sakaiensis* (R103G/S131A mutant; PDB ID: 5XH3_A) and *T.fusca* cutinase TfCut2 (PDB ID:4CG1_A) using Clustal Omega server. The signal peptide of AML (GenBank ID: ADJ49206) was not included in the alignment. The important residues are highlighted as: catalytic residues, substrate binding subsite I, subsite II, extended loop, disulphide bond.

As was found with the sequence alignment, it was evident that the structures of these enzymes overlap with a high degree of similarity (RMSD = 0.472 Å). However, when the wider substrate binding domain was considered (Fig. 8) some subtle divergence was observed. In particularTyr99 of Est119 aligned with Phe110 of AML, and Ile217 with Val226. It is unclear why these differences should account for such divergence in PLA degrading activity given the largely similar nature of the residues. Kitadokoro and co-workers postulated that Tyr99 of Est119 is important as it provides the oxyanion hole to stabilize the esterase reaction [40]. Nonetheless, from this study we can conclude that relatively minor differences in the wider substrate binding site can give rise to significant differences in substrate specificity for plastics.

It was clear that under these mild conditions that AML showed considerable activity towards PBS and PCL but showed no activity toward PLA. While *Amycolatopsis* has been widely reported as having polylactide degrading activity the main enzymes responsible are reportedly proteases – not lipases [36]. Thus, our finding that AML does not degrade PLA is supported by these studies that show that the PLA degrading enzymes secreted by *Amycolatopsis* are proteases [36], [41].

### 3.5 Degradation of aromatic plastics

The most persistent plastics in the environment are the petroleum derived aromatic polyesters, such as PET. These are a significant problem as pollutants and an enzyme capable of degrading this plastic is highly desirable [42]; several studies have attempted PET degradation with varying degrees of success [43].

PET is known to be highly resistant to enzymatic degradation and it is not easily prepared as an emulsion. Therefore, the same analysis could not be used as for the aliphatic polyesters, instead, degradation of the standard PET film was examined via scanning electron microscope (SEM). Degraded PET products (terephthalic acid (TPA) and bis-(2-hydroxyethyl terephthalate; BHET)) in the reaction buffer were explored using high performance liquid chromatography (HPLC) and fluorometric analysis was performed to detect the release of TPA following PET degradation.

Following an extended (96hrs) incubation of AML with PET film, at a moderate temperature (30 °C), there was no observable physical difference in the PET film as determined by SEM (supplementary Fig. 6). Similarly, no PET monomers (TPA or BHET) were detected in the sample buffer after incubation as based on HPLC (supplementary Fig. 7) and fluorometric (supplementary Fig. 8) analyses. Therefore, it was concluded that AML was not capable of observable PET degradation under these conditions. This seemed somewhat surprising since some reports, indicated that PET degrading enzymes (PETases) were cutinases with related structures to AML (see Table 3).

**Fig. 9:**
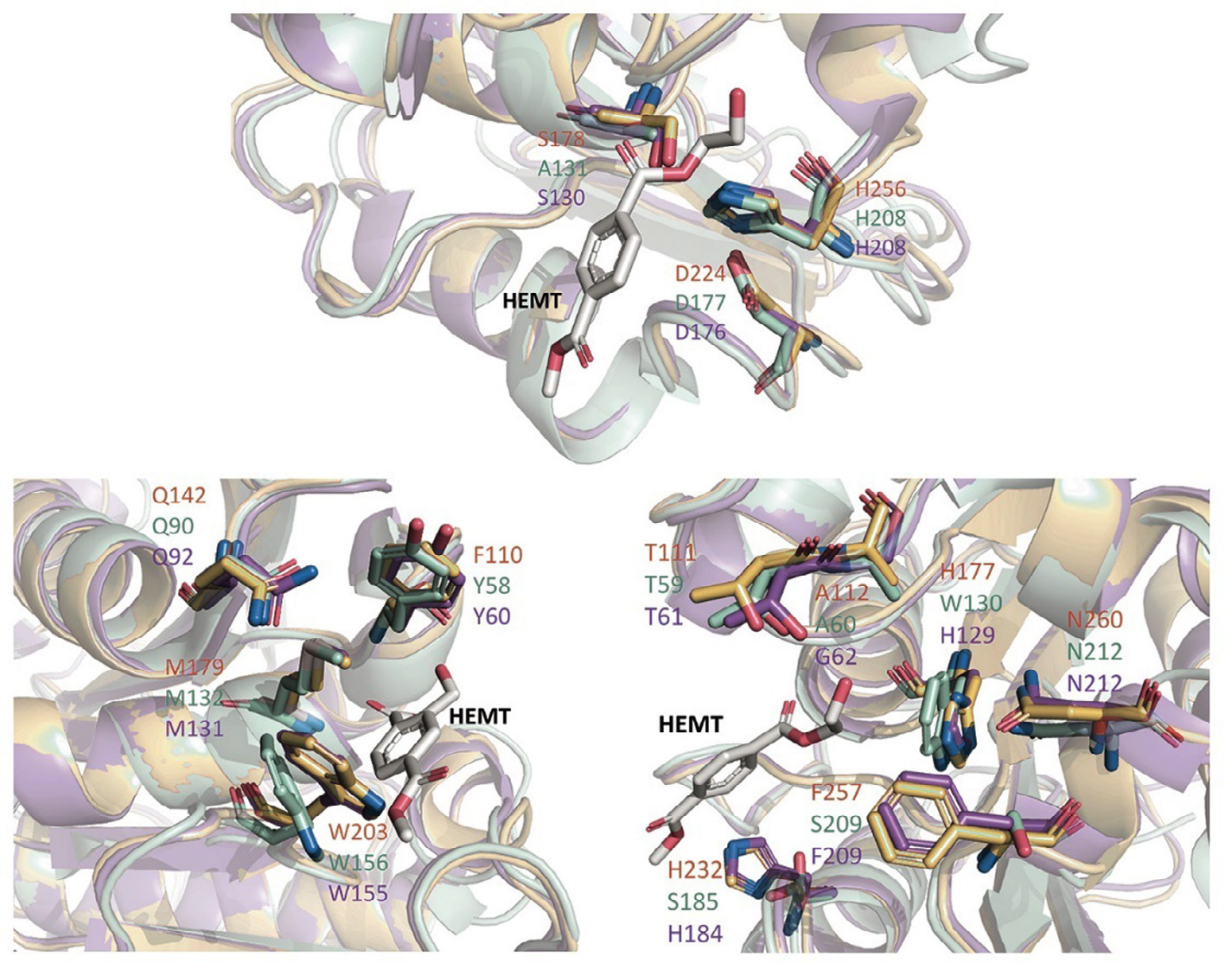
A structural comparison of AML (pale orange) with PETase (pale green; PDB ID: 5XH3; R103G/S131A mutant with 1-(2-hydroxyethyl) 4-methyl terephthalate (HEMT) bound as PET analogue) and TfCut2 (purple; PDB: 4CG1). In the lower images the residues of subsite I (on left) and subsite II (on right) of the active site are shown as stick models. The superposition was performed in PyMol using C_α_ atoms with default parameters. The catalytic triad (Ser-His-Asp) of the enzymes were shown as a stick model while the overall structures were shown as cartoon model. The PET analog HEMT was presented as stick model in white. (For interpretation of the references to colour in this figure legend, the reader is referred to the web version of this article.)

PET degrading enzymes are generally divided into two groups; Type I and Type II based on their binding pocket sequence [44]. The important residues of cutinases involved in PET degradation have been reported previously [44], [45] and this permitted the sequence of AML to be aligned with two known PET degrading enzymes: PETase from *Ideonella sakaiensis* (49% sequence identity, 67% similarity; Type II) [45] and *T.fusca* cutinase (62% sequence identity, 77% similarity; TfCut2, Type I) [28] with the key residues highlighted (Fig. 8). The sequence alignment revealed that AML exhibits matching residues with PET degrading enzymes of Type I. Type 1 enzymes have lower PET degrading activity than Type II [44]. A structural alignment of AML with the structure of PETase from *Ideonella sakaiensis* (PDB ID: 5XH3; with bound 1-(2-hydroxyethyl) 4-methyl terephthalate [HEMT] – a PET analogue) [45] and PET-degrading TfCut2 (PDB ID: 4CG1) [28] was conducted to ascertain their structural similarity. The root mean square deviations (RMSD) of the atomic positions with AML were 0.588 Å and 0.463 Å for PETase and TfCut2 respectively, indicating that there was a high degree of structural alignment between the catalytic residues of AML and the known PET degrading enzyme (Fig. 9). However, some residue differences between AML and the currently known binding pocket features on the PET degrading enzymes were observed (see Fig. 9). Residue differences include S178/A131/S130 in catalytic residues; F110/Y58/Y60 in subsite 1 and in subsite II, A112/A60/G61; H177/W130/H129 and F257/S209/F209 for AML, PETase, TfCut2 respectively. Perhaps, the most significant of these is the F110 in AML, subsite I, which is replaced by a Tyrosine in PETase and TfCut2. The more polar, albeit bulkier, Tyrosine may play a specific role in intermediate stabilisation.

**Fig. 10:**
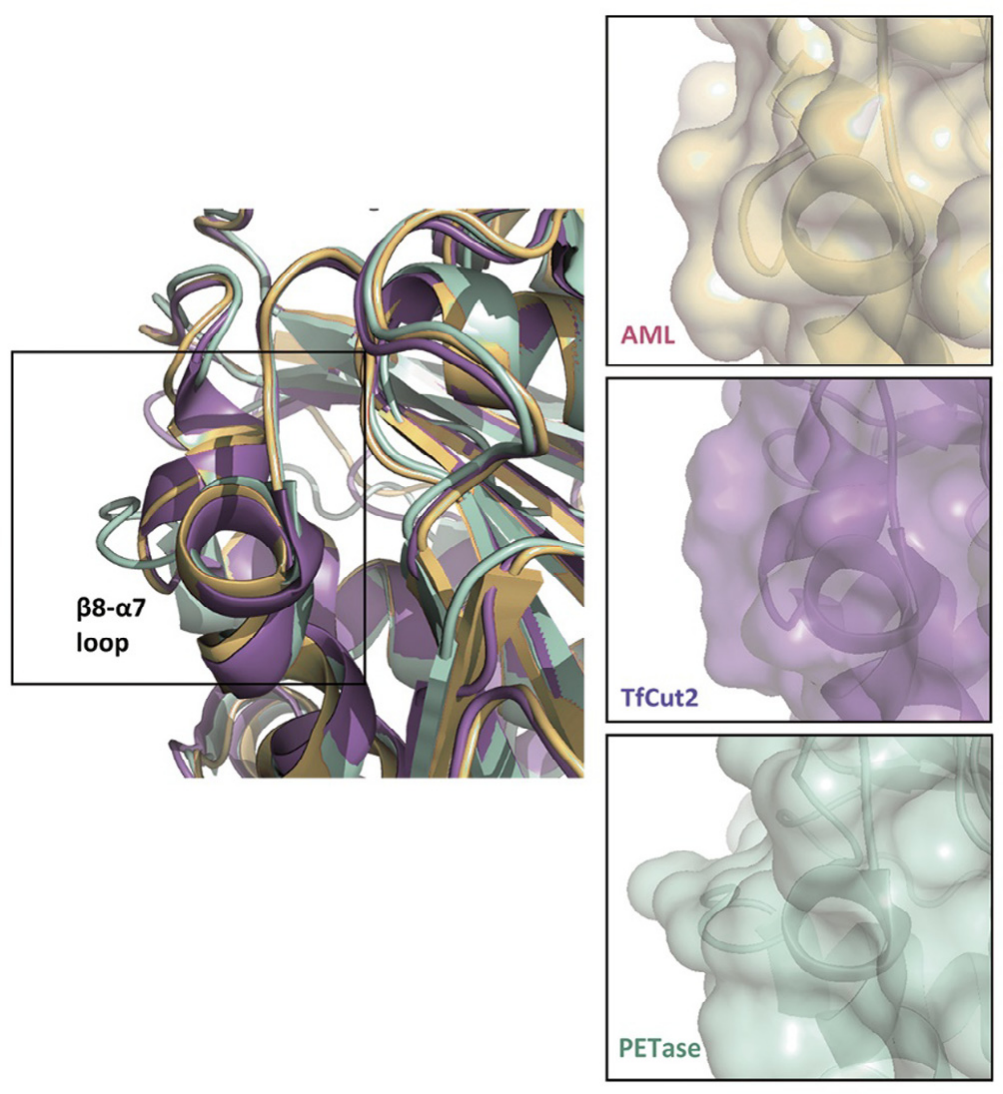
On the left, an overlay structural comparison of AML (mustard) with PETase (pale green; PDB ID: 5XH3; R103G/S131A mutant with 1-(2-hydroxyethyl) 4-methyl terephthalate (HEMT) bound as PET analogue) and TfCut2 (purple; PDB ID: 4CG1) – highlighting the β8-α7 loop region. On the right, are the separated protein surface plots of the same sites. The superposition was performed in PyMol using C_α_ atoms with default parameters. (For interpretation of the references to colour in this figure legend, the reader is referred to the web version of this article.)

A recent paper closely examined the key residues for degradation of PET by a cutinase from *Ideonella sakaiensis* [44]. In a comparison between this enzyme and TfCut2 they showed that the composition of residues in the PET binding subsites I and II could reduce PET binding affinity. Subsite I and II are binding grooves to either side of the catalytic site where PET is bound in extended form to present the scissile bond for hydrolysis. In Fig. 8, we showed that the catalytic triad for AML is the same as for TfCut2. The subsite I binding site differs from that of TfCut2 by having a Phenylalanine (Phe110) in place of a more polar Tyrosine residue while subsite II has a bulkier alanine residue in place of a glycine found in TfCut2. The Phe110 in AML was one of the main differences noted above when comparing PLA degrading activity between AML and Est119 suggesting it may be important in both PLA and PET binding. Whether these differences alone are sufficient to account for the significantly lower PET and PLA degrading activity of AML is unclear given that these are relatively minor changes. This could be explored by appropriate mutagenesis studies. It is worth noting that the wild type TfCut2 had relatively modest PET degrading activity which was subsequently improved by removing bulky residues to allow for binding of extended PET chains [28]. PETase also has an extended section in the β8-α7 loop when compared to AML and TfCut2. This structural difference in the three enzymes, due to the extended loop, was visualised via a surface plot generated using PyMOL (Fig. 10). The extended loop creates an extra groove, possibly for better binding of the polyester, [44] which may partially explain differences in PET activity. It is also worth noting that enzymes capable of efficient PET degradation are not necessarily efficient at degradation of aliphatic polyesters such as PBS or PCL [19].

A report on the commercial application of cutinases in PET recycling published during the study reported here identified leaf-branch compost cutinase (LCC) as highly efficient at PET hydrolysis [61]. Interestingly, these workers found that when 11 key residues in the first contact shell for PET binding were mutated that activity was abolished in most cases. This suggests that minor changes in the configuration of the PET binding site can dramatically influence PET degrading activity which supports the observations noted in our study.

In the latest update of the Lipase Engineering Database v4.0 (https://led.biocatnet.de/), [2] AML (Entry S#455987) is grouped into a Homologous Family #49 with the lipase from *Streptomyces exfoliatus* as the structural representative. The family has 945 protein entries with 1,099 sequence entries and 21 structural entries. As the family also includes most of the reported polyester degrading cutinases and with all the members sharing > 60% sequence homology, structural comparison (besides the substrate binding site) reveals differences between the lipases/cutinases in their ability to degrade different plastic substrates. These include electrostatic and hydrophobicity variations [19], [46] and subtle differences in the extended binding groove [47].

## 4 Conclusion

Homologs of AML were found in several *Amycolatopsis* species indicating that it is probably a key extracellular enzyme of these organisms. Modelling of the 3D structure of AML revealed that the enzyme lacked the lid structure seen with some lipases. This, along with substrate specificity studies, suggested that this enzyme is more appropriately named a cutinase. The catalytic triad residues in AML were identified and homology with known plastic degrading enzymes was observed.

When plastic degrading activity was examined it was clear that the extracellular cutinase from *A. meditarranei* was capable of hydrolysing PCL and PBSc-D but not PLA or PET. A previous report identified that some *Amycolatopsis* species could degrade PLA, PCL and PBS but specific enzymes involved were not isolated [48]. However, studies with *Amycolatopsis orientalis* spp. showed that the extracellular PLA degrading enzymes of these species were proteases and not lipases or cutinases [36]. Our studies support this observation and show that a *single* extracellular enzyme is likely responsible for the degradation of PCL and PBS by *Amycolatopsis* spp. It is clear that *Amycolatopsis* species are capable of degrading a wide range of polyesters and further exploration of this organism in plastics remediation is warranted based on the findings of this study.

Sequence comparison of AML with similar plastic degrading enzymes showed remarkable similarities between these cutinases. The most notable difference was where Phe110 was substituted by a Tyrosine residue in the PLA and PET degrading enzymes examined. Further work is needed to determine if this residue alone plays an important role in binding of PET and PLA.

The specific substrate preferences of AML may be particularly interesting for enzymatic degradation of plastics where recovery of specific monomers is required. Thus, these studies show that PBS and PCL can be preferentially degraded even in the presence of PET and/or PLA under relatively mild conditions. This is potentially useful for degrading mixtures of plastics. Applications in controlled release drug formulations can also be envisaged.

## CRediT authorship contribution statement

**Yeqi Tan:** Conceptualization, Methodology, Software, Validation, Visualization, Investigation, Data curation, Writing - original draft. **Gary T. Henehan:** Conceptualization, Methodology, Supervision, Writing - review & editing. **Gemma K. Kinsella:** Conceptualization, Methodology, Software, Validation, Supervision, Writing - review & editing. **Barry J. Ryan:** Conceptualization, Methodology, Validation, Supervision, Writing - review & editing.

## Declaration of Competing Interest

The authors declare that they have no known competing financial interests or personal relationships that could have appeared to influence the work reported in this paper.
