# Supplementary material for: An extracellular lipase from Amycolatopsis mediterannei is a cutinase with plastic degrading activity
Source: Comput Struct Biotechnol J. 2021 Jan 20;19:869–79. doi: 10.1016/j.csbj.2021.01.019 (PMC7851449; doi:10.1016/j.csbj.2021.01.019)
Supplement: Supplementary data 1 [file mmc1.docx]

Supplementary materials for

***An extracellular lipase from Amycolatopsis mediterannei is a cutinase with plastic degrading activity***

*Yeqi Tan* ^a^*, Gary T. Henehan* ^a^ *, Gemma K. Kinsella* ^a^ *, Barry J. Ryan* ^a, *^

^a^ School of Food sciences and Environmental Health, Technological University Dublin, Grangegorman, Dublin 7, D07 H6K8, Ireland

^*^ ([barry.ryan@TUDublin.ie](mailto:barry.ryan@TUDublin.ie); Ph: 00353-1- 220 5671

ORCID: 0000-0001-7213-3273

| Model | AML (257 residues) | SEL (258 residues) |
| --- | --- | --- |
| Number of residues in stereochemically favoured region (~98% expected) | 252 (98.1%) | 254 (98.4%) |
| Number of residues in allowed region (~2% expected) | 5 (1.9%) | 4 (1.6%) |
| Number of residues in outlier region | 0 (0.0%) | 0 (0.0%) |

**Supplementary figure 1:** Ramachandran plot for AML model (left) and of 1JFR template (right) created using RAMPAGE (Lovell et al., 2003). A comparison of the number of residues in the stereochemically favoured region, the allowed region, and the outlier region are shown in the table.

Residue in allowed region (SEL):

[81:GLY] (102.26, 48.68)

[131:SER] (61.24, -123.35)

[185:HIS] (-122.30, -82.82)

[248:SER] (-152.77, -161.78)

Residue in Allowed region (AML):

[128:GLY] (102.26, 48.68)

[178:SER] (61.24,-123.35)

[209:TRP] (-129.27, 62.67)

[232:HIS] (-122.30, -82.82)

[295:SER] (-152.77,-161.78)


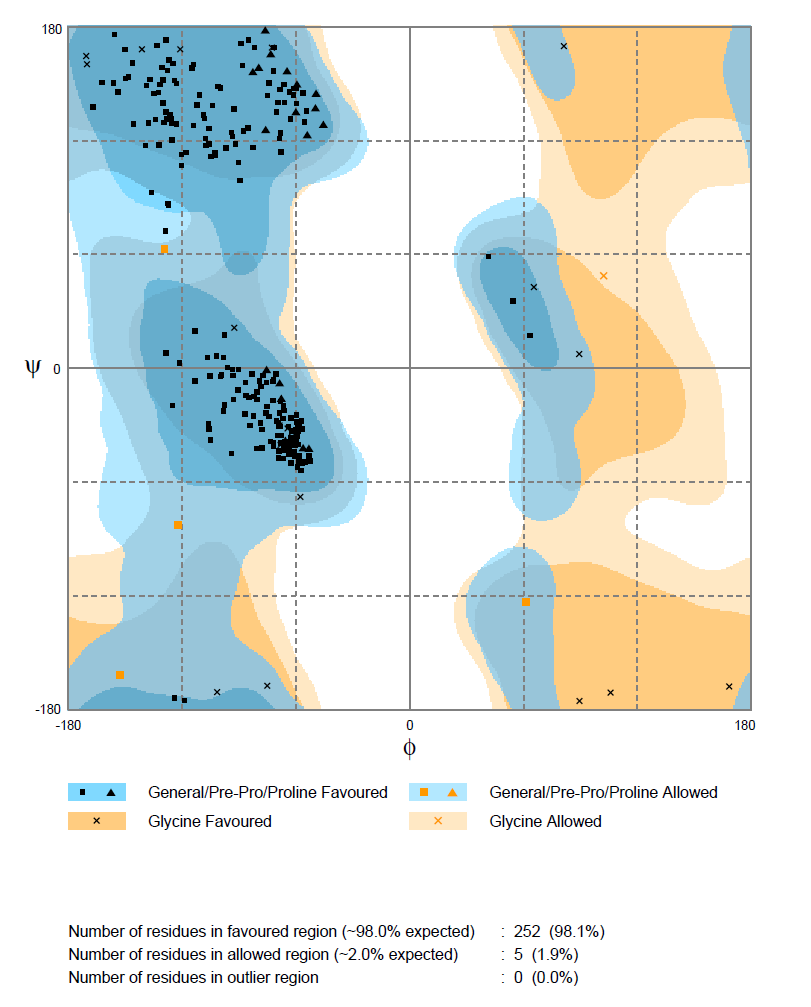

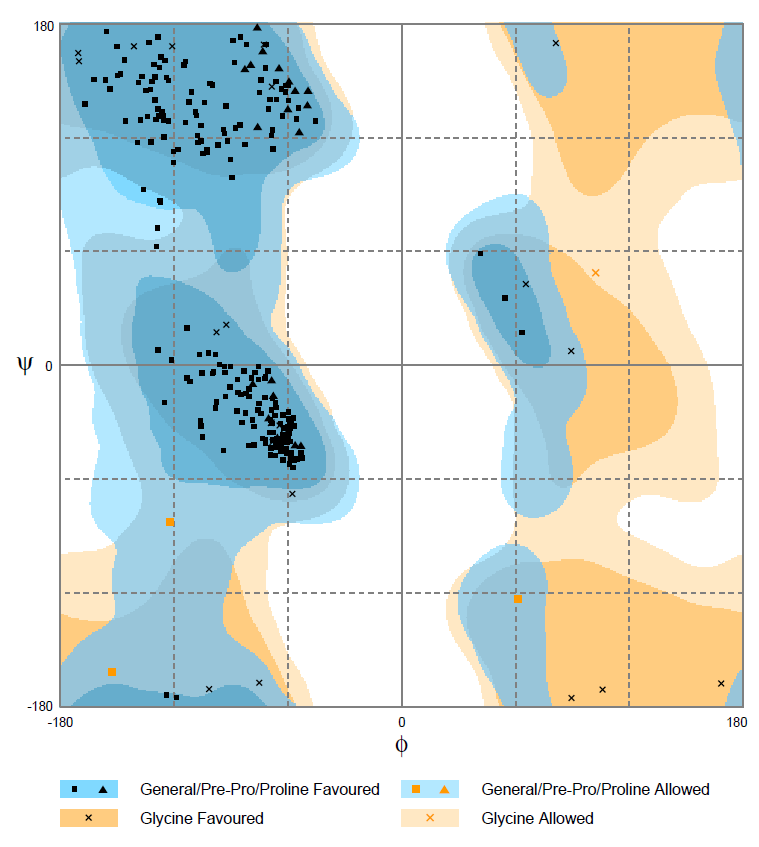


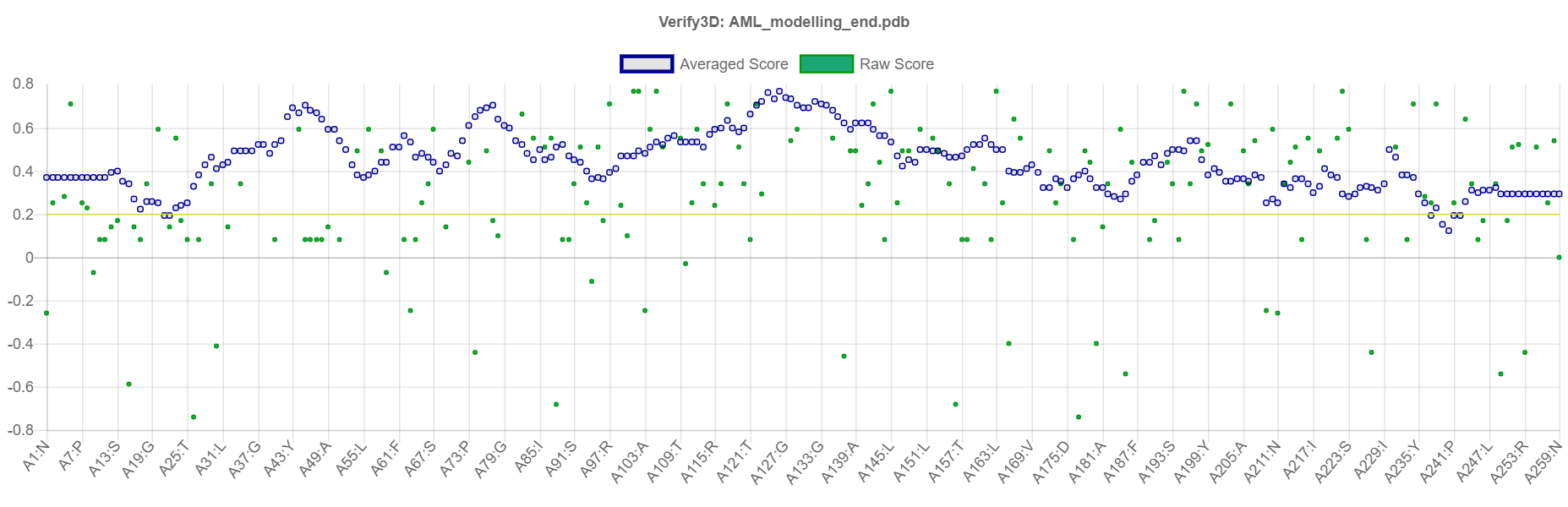


(B)

(A)


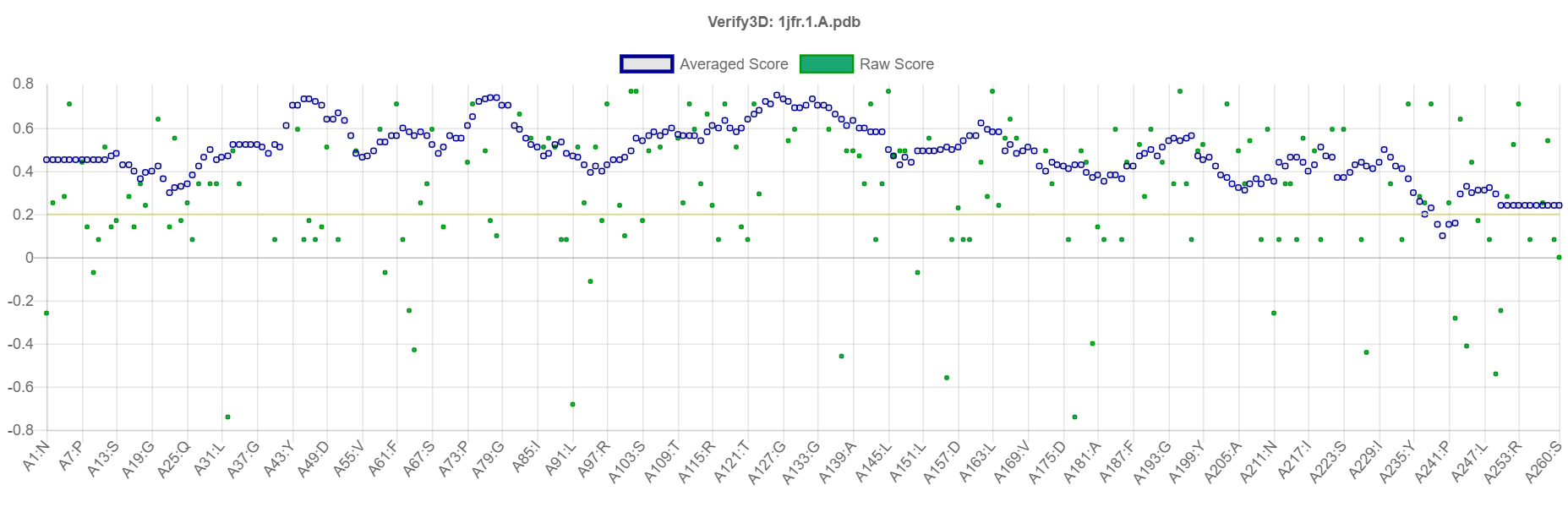


**Supplementary figure 2:** Verify3D plot of (A) AML model and (B) 1JFR model. 97.30% of the AML residues have averaged 3D-1D score >= 0.2 while 98.46% of 1JFR residues have averaged 3D-1D score >= 0.2. The model passes the 3D/1D profile (>80% residues with an averaged 3D-1D score >=0.2).


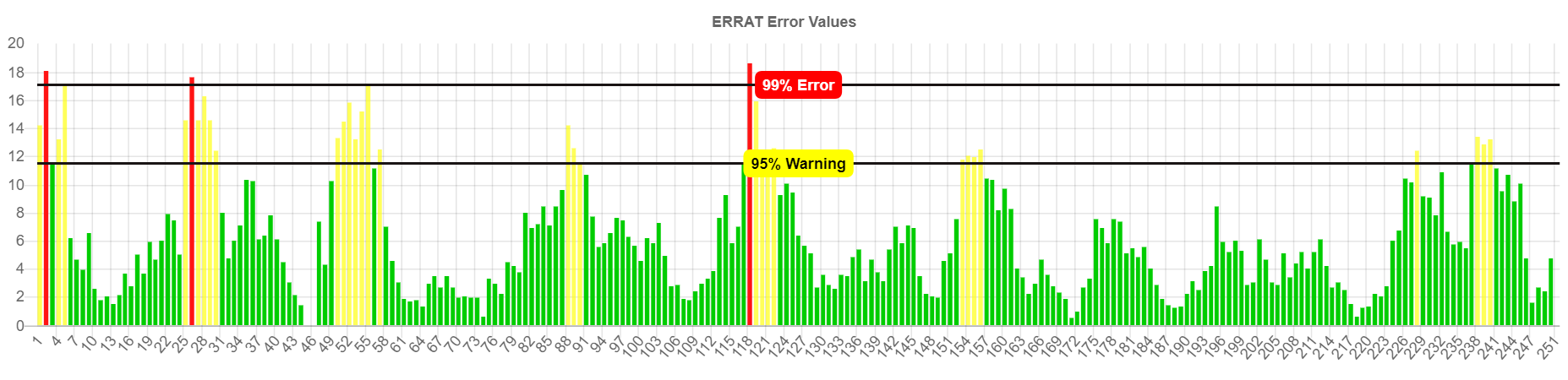


(A)

(B)


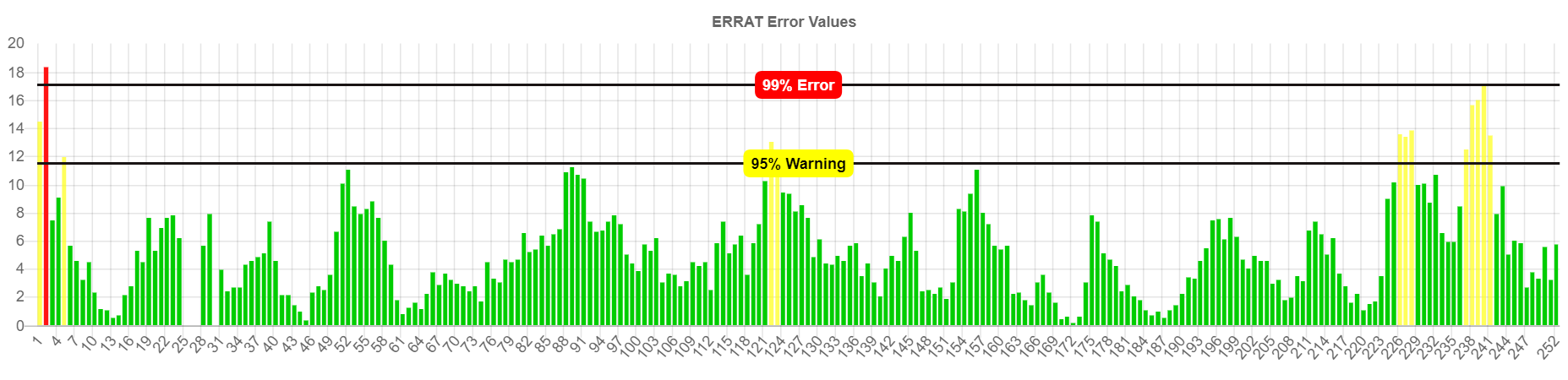


**Supplementary figure 3:** ERRAT plot of (A) AML model (B) 1JFR model. The two black lines indicate the confidence with which it is possible to reject regions that exceed that error value. The percentage of the protein for which the calculated error falls below the 95% rejection limit of the model is of the AML model and 1JFR model are 86.6935 and 94.7581 respectively. A good high resolution structure generally produces value around 95% or higher while lower resolution models (2.5–3Å) has an average overall quality factor around 91%.

(ii)

(i)

(ii)

(i)

(B)

(A)


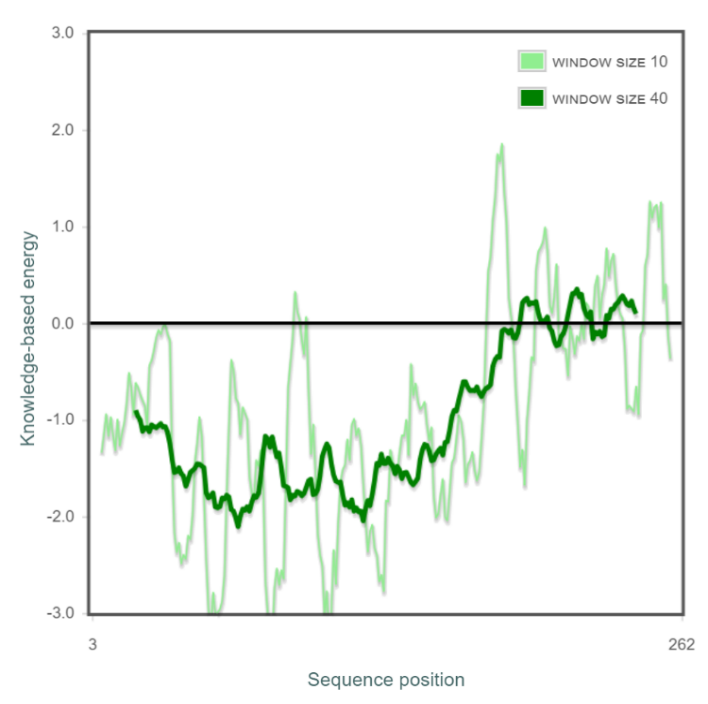

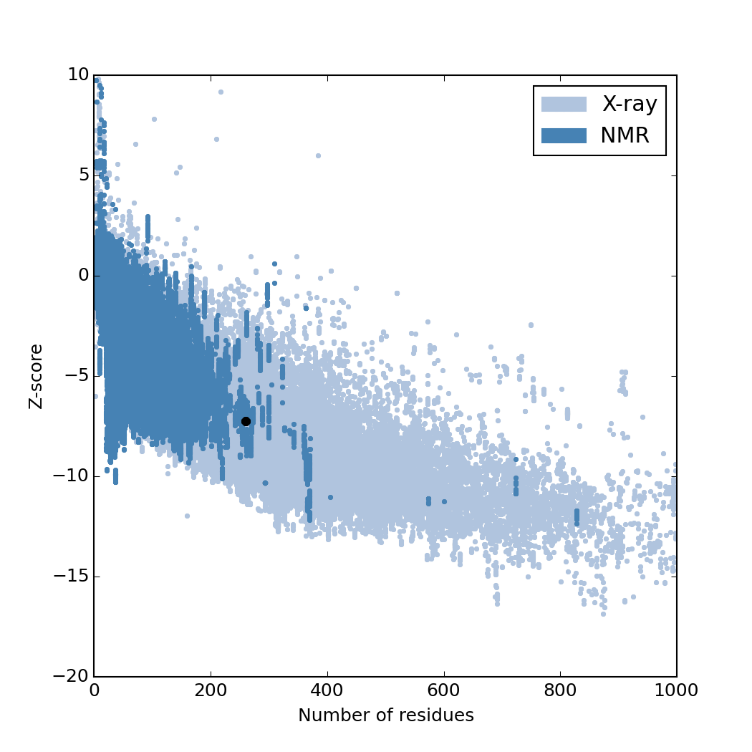

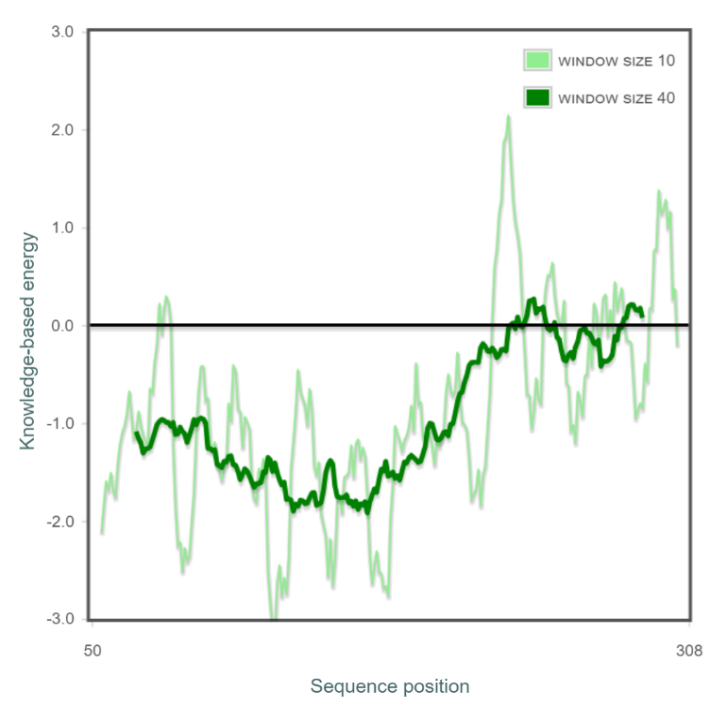

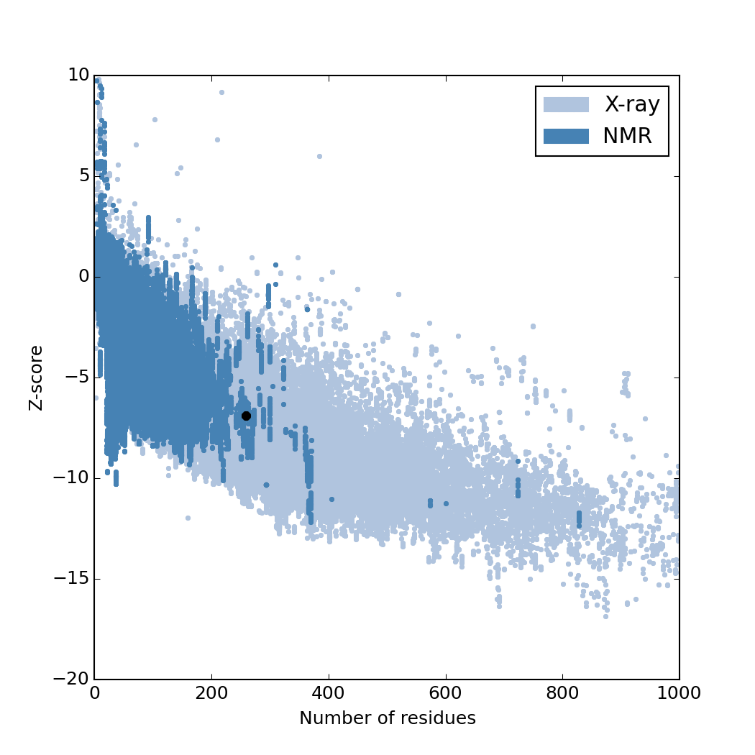


**Supplementary figure 4:** ProSA analysis of (A) AML and (B) 1JFR model. (i) The Z-score of the model is -6.9 and -7.26 respectively - indicated as a black dot (•) on the Z-score plot. The blue data points on Z-score plot are deviations derived from experimentally determined (X-ray and NMR) native structures. A model Z-score outside the experimentally derived range indicates an erroneous structure. (ii) ProSA energy profile of AML and SEL shows the local quality of the model with energy plot as a function of single residue averaged over 40 residues window (Wiederstein and Sippl, 2007).

ADJ49206.1 --MSALTSQPT--SSG-SSEKIPRLRGWRAKAAGVVLAALALTTGVAAPAPAAANPYERG 55

pdb|1JFR|B ----------------------------------------------------AANPYERG 8

********

ADJ49206.1 PDPTTASIEATSGSFATSTVTVSRLAVSGFGGGTIYYPTTTTAGTFGALSIAPGFTATQS 115

pdb|1JFR|B PAPTNASIEASRGPYATSQTSVSSLVASGFGGGTIYYPTSTADGTFGAVVISPGFTAYQS 68

* ** :**** * ::.* .** : .:*******:** * **:**: ::**:** **

ADJ49206.1 SIAWLGPRLASQGFVVFTIDTLTTSDQPDSRGRQLLASLDYLTQQ--SSVRSRIDSTRLG 173

pdb|1JFR|B SIAWLGPRLASQGFVVFTIDTNTTLDQPDSRGRQLLSALDYLTQR--SSVRTRVDATRLG 126

********************* ** ***********::******: ****:*:*.:***

ADJ49206.1 VVGHSMGGGGTLEAARSRPTLQAAVPLTAWDLTKNWSTLQVPTLVVGAQSDTVAPVASHS 233

pdb|1JFR|B VMGHSMGGGGSLEAAKSRTSLKAAIPLTGWNTDKTWPELRTPTLVVGADGDTVAPVATHS 186

*:********:**** ** *:**:*** *. *.* * .***::**: *:*****:*:

ADJ49206.1 IPFYTSLPSTLDRAYLELRGASHFAPNSPNTTIAKYTLSWLKRFIDNDTRYEQFLCPIPS 293

pdb|1JFR|B KPFYESLPGSLDKAYLELRGASHFTPNTSDTTIAKYSISWLKRFIDSDTRYEQFLCPIPR 246

*** *:* : .:********:**:** :******::********.************

ADJ49206.1 TSL--SISDYRGNCPHNG 309

pdb|1JFR|B PSL--TIAEYRGTCPHTS 262

** : :**..***

**Supplementary figure 5:** Multiple amino acid sequence alignment of AML (GenBank ID: ADJ49206.1) with lipases from homologous family abH25.01: SEL (PDB: 1JFR_B). The residues that form the active site (green), the oxyanion hole (magenta) are highlighted in their corresponding colours.


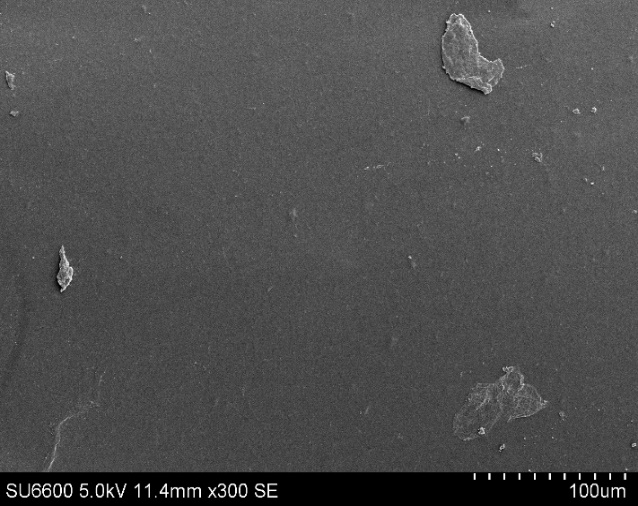

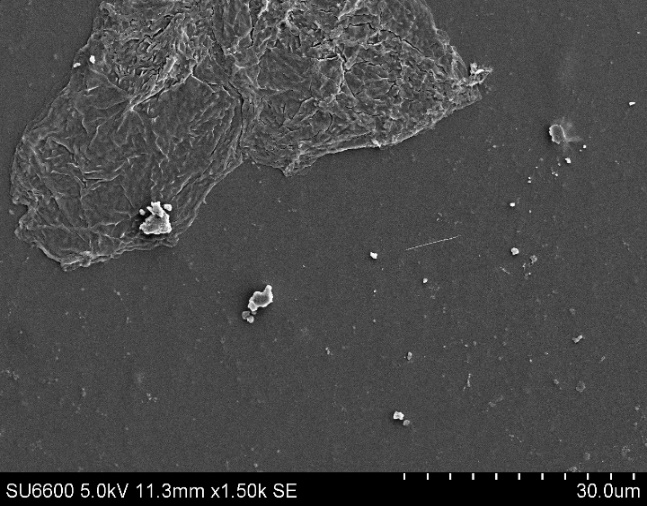

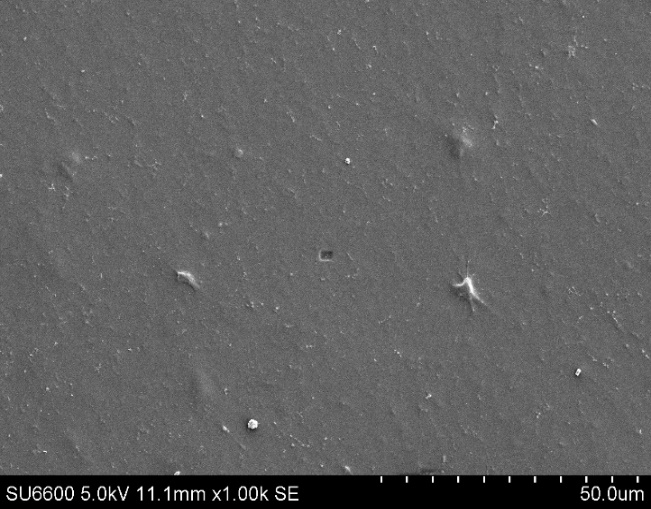

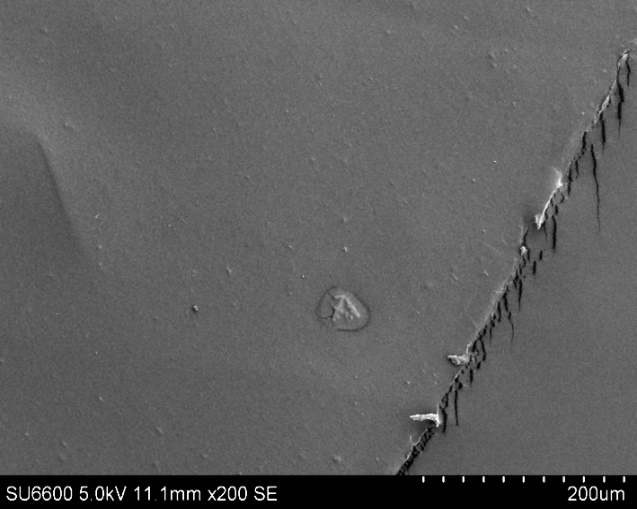


(A)

(B)

**Supplementary Figure 6:** SEM image of PET films (A) non-treated control and (B) AML-treated sample after 96 hr incubation at 30^o^C.

(A)

(B)

**Supplementary Figure 7:** HPLC chromatogram of (A) PET-degradation buffer of AML treated sample and (B) different concentrations of TPA and BHET standards in glycine-NaOH buffer (pH9.0). Peaks with retention time of 3.25 min, 3.5 min and 4.25 min were observed in the standards, but absent in the AML-treated sample.

**Supplementary Figure 8:** Standard curve for the fluorescence emission (Ex/Em=328nm/412nm) of corresponding to the concentration of terephthalic acid (TPA). The experiment was carried out in duplicate.

**Supplementary Table 1:** List of QMEAN score and four of its individual terms (Cβ, all atoms, solvation and torsion) of AML. A value close to zero indicates the property to be similar to the expected value from an experimental structure of similar size. A positive value indicates the model score above the average experimental model while a negative value indicates the model score below the average experimental model.

| **Global quality estimate** | **Value** |
| --- | --- |
| **QMEAN** | -0.46 |
| **Cβ** | 0.19 |
| **All atom** | -0.90 |
| **Solvation** | -0.74 |
| **Torsion** | -0.22 |

**Supplementary Table 2:** Results table of the fluorescence based detection of TPA released from enzymatic PET hydrolysis. The sample was treated with AML at 30^o^ for 72 hr and the control was PET incubated without AML as described in Section 2.2.30 (p.120)

| **TPA conc (mM)** | **Reading 1** | **Reading 2** | **Average** | **Corrected** | **Standard deviation** |
| --- | --- | --- | --- | --- | --- |
| **0** | 2.293 | 2.536 | 2.415 | 0.000 | 0.1215 |
| **0.006** | 2.643 | 2.773 | 2.708 | 0.294 | 0.065 |
| **0.03** | 5.118 | 5.295 | 5.207 | 2.792 | 0.0885 |
| **0.06** | 8.698 | 8.389 | 8.544 | 6.129 | 0.1545 |
| **0.12** | 12.64 | 12.12 | 12.380 | 9.966 | 0.26 |
| **0.18** | 17.85 | 20.55 | 19.200 | 16.786 | 1.35 |
| **Sample 1** | 2.210 | 1.753 | 1.982 | -0.433 | 0.2285 |
| **Sample 2** | 2.242 | 1.795 | 2.019 | -0.396 | 0.2235 |
| **Ctrl** | 1.776 | 1.976 | 1.876 | -0.539 | 0.1 |
